# Low level of awareness and prevention of hepatitis B among Congolese healthcare workers: urgent need for policy implementation

**DOI:** 10.3389/fpubh.2024.1463455

**Published:** 2024-10-08

**Authors:** Tony Akilimali Shindano, Yves Horsmans

**Affiliations:** ^1^Université Catholique de Bukavu, Bukavu, Democratic Republic of Congo; ^2^Hôpital Provincial Général de Référence de Bukavu, Bukavu, Democratic Republic of Congo; ^3^Department of Internal Medicine, University of Kindu, Kindu, Democratic Republic of Congo; ^4^Center for Tropical Diseases and Global Health, CTDGH, Bukavu, Democratic Republic of Congo; ^5^Cliniques Universitaires Saint-Luc, Woluwe-Saint-Lambert, Belgium

**Keywords:** hepatitis B, policy, prevention, health workers, DRC

## Abstract

The Democratic Republic of Congo (DRC) is a country with many public health challenges, including those related to the prevention and management of viral hepatitis B. Healthcare workers, who are at the frontline of patient care, are particularly at risk of contracting and spreading this virus, especially given its high prevalence in the general population. This paper examines the level of awareness and preventive measures among Congolese healthcare workers. Overall, the data show that health workers are under-immunized and lack formal training in hepatitis B prevention and management. In addition to limited awareness, health facilities are insufficiently involved in the implementation of standardized infection control protocols, the provision of personal protective equipment and routine hepatitis B vaccination programmes. There also appears to be a lack of clear and effective national policies outlining the main axes of infection control targets by 2030. This calls for urgent policy implementation focusing on mandatory vaccination, training, resource availability, adherence to infection control practices and comprehensive post-exposure management.

## Introduction

Hepatitis B virus (HBV) infection is a major global health problem. According to the World Health Organization (WHO), hepatitis B accounts for 3.8% of the global burden of disease. In 2019, it was estimated that 296 million people were living with chronic hepatitis B, including 1.5 million of newly infected patients (1). Approximately 820,000 deaths were attributed to HBV-related complications, including cirrhosis and hepatocellular carcinoma (1, 2).

Hepatitis B is hyper-endemic in Africa and it is estimated that more than 60 million chronic carriers live in this part of the world (3). However, there is considerable variation between regions. Regions of intermediate prevalence have rates between 2% and 8% and include the Democratic Republic of Congo (DRC) (4–7).

HBV is a communicable disease that is transmitted by contact with the blood or other body fluids of an infected person. Several populations are considered to be at high risk of acquiring the infection. Among these, health care workers (HCWs) are prominent because of the risk of occupational hazards associated with exposure to infectious blood or body fluids (8, 9). Studies have shown that the risk of acquiring hepatitis B is estimated to be up to four times higher than in the general population (8). The risk is even greater because transmission can occur through minimal or ignored lesions (9). Unfortunately, common safety precautions are often ignored, and even when they are known, they are sometimes deliberately neglected.

One of the simplest and most effective methods of protection is vaccination. As one of the tools in plans to eliminate viral hepatitis worldwide, it has been consistently recommended by the WHO for all high-risk groups, especially HCWs (3). In several African countries, efforts are currently being made to make it mandatory only for newborns, although there are still disparities in vaccination coverage.

Unfortunately, several studies have also shown very low vaccination coverage among African HCWs compared with other continents (10).

Several reasons have been identified, including not only a lack of policy, vaccine supply and information, but also cultural barriers, self-financing and the trivialization of the disease (9).

## Epidemiology of hepatitis B in the DRC and vaccination strategy

### Epidemiology of hepatitis B

The DRC is currently considered as a country of intermediate endemicity. Recent data, including one of our works, estimate the prevalence of hepatitis B surface antigen to be between 3.9% and 5% (5–7). Since 2019, the hepatitis B vaccine has been included in the national Expanded Programme on Immunization, which consists of 3 doses, the first of which is administered after 6 weeks of age (11).

Interestingly, the data show a lower prevalence than previously reported, probably related to the effect of neonatal vaccination for almost 15 years (5–7). Unfortunately, the country has not yet implemented a systematic birthday dose and extension to other risk groups, contrary to recommendations. However, since the introduction of the vaccine, coverage has remained relatively low. The most recent rate is between 60% and 70% (12), leaving a large proportion of the population at risk unvaccinated and exposed to HBV.

### HCW and vaccination coverage

HBV is a highly contagious blood-borne infection that can be transmitted through percutaneous injuries or medical procedures. This situation puts HCWs at very high risk of contamination due to numerous potential occupational hazards. This is even more serious in the African context, which is characterized by a high prevalence of hepatitis B combined with a lack of adequate protective measures.

It is estimated that about one-third of HCWs in Africa are exposed annually to the risk of occupational exposure to blood or other body fluids or after percutaneous injuries (13).

In this context, vaccination against HBV remains the only and safest means of protection. Unfortunately, in many countries, vaccination policies have yet to be implemented. Several studies have reported low coverage of hepatitis B vaccination among HCWs in Africa. This rate is even lower in the DRC than in Libya, for example, where coverage of 72% has been reported (10, 14). In fact, a recent meta-analysis showed that the DRC had one of the lowest HBV vaccination coverage rates among HCWs in Africa (10). Studies have shown that coverage of 3 doses of HBV vaccine varies from 0.9% in Bukavu to 4.2% in Butembo, two cities in eastern DRC (15, 16).

Studies have also shown that occupational blood exposure accidents are very common in the country. Among the available data, one of our studies reported 30–40% of accidents during medical practices (16, 17).

Very few HCWs are aware of the risk of contamination. This recent situation is very alarming.

## Discussion

### Prevention of HBV infection among HCWs

The prevention strategy should be based on three levels: one personal, and two institutionals. It therefore involves compliance with standard health care precautions as well as active immunization strategies (vaccination) or passive in the context of post-exposure prophylaxis. The tertiary level involves public authorities through the implementation of national policies.

### Primary prevention level

This level implies that all HCW must consider their workplace as a risk and that any biological product may contain transmissible infectious agents until proven otherwise. In this context, the most dangerous blood-borne pathogens are HIV and hepatitis.

Standard precautions include hand hygiene, wearing gloves, a mask or face shield, laboratory coats and other protective clothing. Other precautions include systematic hand washing before and after all contact with patients or their body fluids, and proper cleaning and disinfection of all body surfaces exposed to blood or body fluids.

Particular attention should be paid to all high-risk practices, such as injection practices. HCWs should be made aware of the need to favor the use of single-use sharp instruments, to dispose of used contaminated sharp instruments in puncture-resistant containers and to avoid recapping used needles. Further efforts need to be made in this area because, for example, not only are blood exposure accidents numerous, but most HCWs still engage in risky behaviors such as bare-handed phlebotomy and recapping of needles during care (16). This could be mainly related to working environment issues, but also to inadequate training and awareness.

### Secondary prevention

This is the institutional level of prevention, which includes the effectiveness of a local training programme, a good working environment, an appropriate work schedule, a staff vaccination programme, and the management of blood exposure accidents and HBV-infected HCWs.

Of all these measures, routine vaccination of HCWs is the most important. In fact, WHO recommends that all groups with occupational exposure as HCWs should be considered as one of the primary targets for vaccination. Similarly, both ensuring that HCWs are adequately vaccinated (targeting the protective anti-HBs level of ≥10 mIU/mL) and monitoring their immune status are recommended.

Unfortunately, in the DRC context, many facilities are unable to meet this requirement due to lack of funding. The data show that many HCWs are not properly vaccinated for a variety of reasons, mainly ignorance and a self-sustaining system of vaccination.

Very few facilities have a blood exposure accident management programme or staff retraining programme. This may explain the low level of knowledge among HCWs about blood-borne diseases in general and hepatitis in particular. In addition, this not only exposes HCWs to risky behaviors, but also increases the risk of acquiring hepatitis after exposure.

Facilities should also be involved in the management of their infected staff. This should help not only to prevent complications, but also to reduce the risk of direct transmission to patients during care. Unfortunately, there is currently little data on the prevalence of viral hepatitis among HCWs in the DRC.

### Tertiary prevention

In 2016, the World Health Assembly, of which the DRC is a member, adopted a comprehensive strategy to eliminate hepatitis B and C by 2030. This plan has several axes, the most important of which is the need for each Member State to implement a national plan and clear policies to achieve this goal. One of the priority actions for countries should focus on occupational health, and on this point the Assembly set the implementation of routine hepatitis B vaccination among high-risk groups, including HCWs, by 2020 as one of the goals of this strategy.

In fact, the DRC is lagging behind this plan. Although a national routine childhood immunization programme has been in place for more than a decade, coverage is still not optimal (12). In addition, the introduction of the vaccine at birth is not yet effective.

However, progress has been made with the launch of a national hepatitis programme. However, there is still no clear plan or national policy on hepatitis. Given the prevalence of hepatitis, the population density and the large number of health facilities and medical and nursing schools, urgent action is needed.

As a concrete action proposal, the country should make the vaccination of HCWs mandatory and systematic. This should include all preclinical medical and nursing students. One way of operationalizing this proposal would be to include the cost directly in school fees.

In addition, the government should strengthen education programmes on infection control and help health facilities to implement recycling programmes and internal management programmes for occupational exposures.

Finally, a national surveillance and reporting system should be established. This would help to better identify progress made and efforts still to be made.

## Conclusion

The DRC has endorsed the global strategy to eliminate hepatitis B and C by 2030. This programme includes strategies targeted at HCWs, as they are considered a risk group. The current situation shows that several actions need to be taken to achieve this goal on time. Urgent strengthening of the education system, improvement of the working environment, institutional support and a national policy including routine vaccination of HCWs are needed.
